# ABSTRACTS FROM THE SECOND ANNUAL RESEARCH DAY HOSTED BY THE MICHIGAN STATE UNIVERSITY COLLEGE OF OSTEOPATHIC MEDICINE, NOVI, MICHIGAN, APRIL 11, 2024.

**DOI:** 10.51894/001c.122744

**Published:** 2024-08-30

**Authors:** Andrea Amalfitano, Patricia Obando, Rana Ismail, Francis Akenami

**Affiliations:** 1 College of Osteopathic Medicine Michigan State University

**Keywords:** Abstracts, Michigan State University College of Osteopathic Medicine (MSUCOM), Research Day, Clinical, Basic science, Medical education, Quality improvement, Publication, Manuscripts, Spartan Medical Research Journal (SMRJ)

## TABLE OF CONTENTS

**ABSTRACTS FROM THE SECOND ANNUAL RESEARCH DAY HOSTED BY THE MICHIGAN STATE UNIVERSITY COLLEGE OF OSTEOPATHIC MEDICINE, NOVI, MICHIGAN, APRIL 11, 2024**. **Andrea Amalfitano, DO, PhD**, Patricia Obando, PhD; Rana Ismail, PhD, MSc, CPHQ; Francis Akenami, BMLS, PhD, MSc, FIMLS.

**DEVELOPMENT OF AN SRF INHIBITOR PEPTIDE FOR THE TREATMENT OF CARDIAC FIBROSIS. Melissa Meschkewitz, BSc (DO/PhD Student);** Erika Lisabeth, PhD (Faculty); Richard Neubig, MD, PhD (Faculty).

**DYNAMIC EFFECTS OF DIETARY PRUNE ON THE GUT-BONE AXIS IN HEALTHY FEMALE MICE. Nicholas J. Chargo, BS (DO/PhD Student)**; Kavisha Patel, MS (Research Assistant); Shashank Ravishankar (Undergraduate Student); Leon Regnery (Undergraduate Student); Douglas V. Guzior, MS (Graduate Student); Joseph D. Gardinier, PhD (Faculty); Robert A. Quinn, PhD (Faculty); Narayanan Parameswaran, BVSc, PhD (Faculty); Laura R. McCabe, PhD (Faculty).

**FULL DOSE CHALLENGE OF MODERATE, SEVERE, AND UNKNOWN BETA-LACTAM ALLERGIES IN THE EMERGENCY DEPARTMENT. Stephanie Coallier, MD (Resident)**; Reid Mitchell, DO (Resident); Adam M. Anderson, MD (Faculty); Lisa E. Dumkow, PharmD (Faculty); Lauren M. Wolf, PharmD (ED Pharmacist).

**EXPLORING TYPE I INTERFERON SIGNALING AS A TRANSLATIONAL TARGET IN PVT1 EXON 9 OVEREXPRESSED NEUROENDOCRINE PROSTATE CANCER. Rachel E. Bonacci, PhD (Post-doc Researcher)**; Meghan McGill; Colin Finnegan; Chinedum Udekwu, MS (Graduate Student); Baris Boylu, PhD; Olorunseun O. Ogunwobi, MD, PhD (Faculty).

**A NOVEL PREOPERATIVE CALCULATOR FOR PERIPROSTHETIC JOINT INFECTION FOLLOWING TOTAL JOINT ARTHROPLASTY BASED OFF THE MARCQI DATABASE. Daniel Gerow, DO (Resident)**; Huiyong Zheng, PhD (Faculty); Richard Hughes, PhD (Faculty); Kory Johnson, DO (Faculty); Brian Hallstrom, MD (Faculty).

**A GENOME-WIDE CRISPR/CAS9 SCREEN REVEALS EPIGENETIC IMMUNE EVASION MECHANISMS IN HUMAN PAPILLOMAVIRUS-POSITIVE HEAD AND NECK SQUAMOUS CELL CARCINOMA. Nicholas S. Giacobbi (DO/PhD Student)**; Lexi Vu (Graduate Student); Shreya Mullapudi (Medical Student); Canchai Yang; Niklas Alexander Wahl (Undergraduate Student); Mohamed Khalil, PhD (Faculty); Andrew Olive, PhD (Faculty); Dohun Pyeon, PhD (Faculty).

**CORRELATES OF DEPRESSION/ANXIETY AND LITERACY IN FEMALE CAREGIVERS LIVING WITH HIV IN UGANDA AND MALAWI**. **Tiffany Zheng, BSc (Medical Student)**; Jenus Shrestha (Research Assistant); Itziar Familiar-Lopez, MD PhD (Faculty).

**INTERPROFESSIONAL EDUCATION (IPE) EVENT DEVELOPS COLLABORATIVE COMPETENCIES IN OSTEOPATHIC MEDICAL STUDENTS AT MICHIGAN STATE UNIVERSITY**. **Aastha Bahl (Medical Student);** Carolina Restini, PharmD, PhD (Faculty); Lauren Azevedo, DO, MS, ABP (Faculty); Jackeline Iseler, DNP, MSN, RN, ACNS-BC, CNE (Faculty); Stephanie M. Jalaba, MMS, PA-C (Faculty); Scott Carlson, DO, MPH (Faculty); Gregory J. Spray, PhD, CCC-SLP (Faculty); Fabrice Mowbray, PhD, RN (Faculty); Kirsten Waarala, DO, FACOI, FAODME (Faculty).

**RARE CASE OF CAROLI DISEASE WITH CHOLEDOCHODUODENAL FISTULA AND SUMP SYNDROME. Pujyitha Mandiga, MD (Fellow);** Rene Peleman, MD (Faculty).

**LOW RISK CHEST PAIN FOLLOW UP: A CASE SERIES. Nick Stiles, DO (Resident);** Virginia Labond, MD (Faculty).

**DEVELOPMENT OF A BIOMARKER TEST FOR TIGIT IN A DIVERSE COHORT OF HUMAN PANCREATIC CANCER PATIENTS. Madison George (Medical Student);** Julie Clark, PhD (Faculty); Kendyll Gartrelle (Medical Student); Georges Nassif, MD (Research Fellow); Kailee Hartway (Graduate Student); Daniel Long (Histology Technician); Daniel J. Sålas-Escabillas (Graduate Student); Allison Wombwell (Histology Technician); Hui-Ju Wen, PhD (Research Investigator); Simone Benitz, PhD (Postdoctoral Researcher); Samuel Zwernik (Research Coordinator); Rupen Shah, MD (Faculty); Hakmin Park, MD (Faculty); Philip A. Philip, MD (Faculty); Gazala Khan, MD (Faculty); Howard Crawford, PhD (Faculty); David Kwon, MD (Faculty); Brian Theisen, MD (Faculty); Nina G. Steele, PhD (Faculty).

**MANAGEMENT OF A PEDIATRIC LYMPHANGIOMA ON THE BUTTOCKS. Victoria Qian, BSc(Medical Student);** Michael Davis, DO (Resident); Chaya Pitman-Hunt, DO (Faculty).

**UNUSUAL EXTERNAL BLOCKAGE - NECK ABSCESS LEADING TO EXTERNAL JUGULAR VEIN THROMBOSIS: AN UNCOMMON COMPLICATION. Shamaiza Waqas, MD (Resident);** Khin Pyai, MD (Resident); Waqas Abid, MD (Faculty).

**FEASIBILITY OF USING ASPEN BRACES TO IMPROVE POSTURE IN PISA SYNDROME ASSOCIATED WITH PARKINSON’S DISEASE: A CASE REPORT. Cirus K. Shiran, BSc (Medical Student);** Benjamin M. Roskiewicz, BSc (Medical Student); Jacek Cholewicki, PhD (Faculty); Angela S. Lee, MPH (Research Assistant); John M. Popovich Jr., PhD, DPT (Faculty); John L. Goudreau, DO, PhD (Faculty).

**TIA OR ANOTHER CHAMELEON? Beom Ho (Daniel) Lee, BSc (Medical Student);** Malitha Hettiarachchi, MD, FACP (Faculty).

**FLINT TOOTH FAIRY (FLINT ASSESSMENT OF IN-UTERO AND AT-RISK YOUNG): THE APPLICATION OF NOVEL DENTITION ANALYSIS TO UNDERSTAND HISTORIC LEAD EXPOSURE. Jasmin Cambri, BS (Medical Student);** Mallory Goldsworthy, MPH (Faculty); Danielle Land, PhD (Faculty); Jenny LaChance, MS, CCRC (Faculty); Mona Hanna-Attisha, MD, MPH (Faculty).

**HYPERLACTATEMIA IN SEPTIC SHOCK, UNMASKING THE FALSE NOTION: AN INTRIGUING CASE REPORT. Mehwish Zeb, MD (Resident);** Apoorv Tiwari, MD (Resident); Mazen Hasan, DO (Resident); Abed Dabaja, MD (Faculty); Sujata Kambhatla, MD (Faculty).

**AN EPIDEMIOLOGICAL REVIEW OF OCULOFACIAL TRAUMA WITHIN A DETROIT HOSPITAL SYSTEM. Deanna Ingrassia Miano, DO (Resident);** Parker Williams, DO (Resident); Jeff Kuhary, DO (Resident); Neena Singhal, BS (Medical Student); Mohamad Haidar, DO (Resident); Aishwarya Bhan, DO (Resident).

**PREVALENCE OF AND ATTITUDES TOWARD MARIJUANA: THE CASE FOR CHALDEAN AMERICANS IN MICHIGAN. Anthony Mansour, BSc (Medical Student);** Anthony Cholagh (Medical Student); Bianca Elias (Medical Student); Angelina Selou, BS; Carrie Nazaroff, PhD; Florence Dallo, PhD, MPH (Faculty).

**DIDN’T SEE THIS COMING – DIAGNOSIS OF OCULAR SYPHILIS IN THE EMERGENCY DEPARTMENT. Irene Young, DO (Resident);** Christopher Nedzlek, DO (Faculty).

**NON-ARTERITIC ANTERIOR ISCHEMIC OPTIC NEUROPATHY SECONDARY TO TADALAFIL USE. Paige Mellema, BSc (Medical Student);** Blake Deutmeyer (Medical Student); Paul Wasielewski MD (Faculty).

**SMALL INTESTINE GASTROINTESTINAL STROMAL TUMOR MASQUERADING AS UTERINE MALIGNANCY. Brandon Wiggins, DO, MPH (Fellow);** Mark Rigby, DO (Resident); Elaine Ognjanovsk, (Medical Student); Mark Minaudo, DO (Faculty).

**REDUCING LAPSES IN HEMOGLOBIN A1C TESTING. Brandon Elliott, MD (Resident);** Lisa Wade, DO (Faculty).

**MOLECULAR BASIS OF CLEFT PALATE IN A SPONTANEOUS DOG MODEL. Justina Kwaghe, BSc (Medical Student);** Madelyn Jeffrey (Medical Student); Saira Malik (Medical Student); Brian C. Schutte (Faculty).

**IMPORTANCE OF PROMPT CREATINE KINASE MONITORING IN DIABETIC EMERGENCIES. Apoorv Tiwari, MD (Resident);** Melanie Corsten, PA; Syeda Salari, MD (Resident); Nikhale Malik, MD (Resident); Abed Dabaja, MD (Faculty); Chadi Saad, MD (Faculty).

**CASCADING IMPACT: LEVERAGING MEDICAL STUDENTS AS CATALYSTS FOR STOP THE BLEED TRAINING IN COMMUNITIES. Elizabeth N Montgomery, EMT-B (Medical Student);** Bret Bielawski, DO, FACP (Faculty).

**GENDER-AFFIRMING SCROTECTOMY: INITIAL DESCRIPTION AND OUTCOMES. Kitan Zoltin, MS (Medical Student);** Briar Shannon, RN (Nurse Navigator); Ryan Timar, MD (Resident); Nabeel Shakir, MD (Faculty).

**GASTROINTESTINAL MYELOID SARCOMA: A CASE REPORT. Jacob Burch, DO (Fellow);** Fady Banno, MD, MSc (Resident); Justin Miller DO (Faculty).

**RUPTURED SPLENIC ABSCESS WITH PNEUMOPERITONEUM SECONDARY TO CHRONIC PYELONEPHRITIS. Erica Roach, BSc (Medical Student);** Hutton White, MD (Resident); Jacob Schaub, DO (Resident); Kyle Moehlin, DO (Resident); Michael Kopek (Medical Student); Tarik Wasfie, MD FACS (Faculty).

**IMPROVING COMFORT LEVELS IN PERFORMING AWAKE FIBEROPTIC INTUBATION: A COLLABORATIVE EFFORT BETWEEN OTOLARYNGOLOGY AND ANESTHESIA. Benjamin Gillette, DO (Resident);** Michael Toscano, DO (Resident); Michael Owens, MD (Faculty); Gary Kwartowitz, DO (Faculty).

**RENAL INFECTION VS INFARCTION: A CHALLENGING DIAGNOSIS AND A RARE CASE REPORT. Spencer Gilbert BS (Medical Student)**; Bashar Hammad MD (Resident); Heba Al Sabbagh MD (Resident); M. Ammar Hatahet MD, MPH, FACP (Faculty).

**SURVIVAL TREND IN COLO-RECTAL CANCER IN AFRICAN AMERICAN PATIENTS OVER THE LAST DECADES. Vihangi Niyangoda, MS (Medical Student)**; Tarik Wasfie, MD (Faculty); Hemal Dholakia (Medical Student); Erica Roach (Medical Student); Harold Freeman, MD (Faculty).

**ASSEMBLY LINE AILMENT: A TALE OF A DISCOLORED DIGIT IN THE AUTO INDUSTRY. Konstantin Rosich, DO (Resident);** Christopher Nedzlek, DO (Faculty).

**TRENDS IN REPEAT LACTIC ACID TIMING AND PATIENT OUTCOMES IN A COMMUNITY HOSPITAL IN THE MIDWEST. Jordan Place, DO (Resident);** Virginia Labond, DO (Faculty).

**RING RASH IN AN INFANT: UNMASKING NEONATAL LUPUS. Aashir Siddique, BSc (Medical Student);** Kripa Thakur, MD (Faculty); Landon Barlow, BSc (Medical Student), Michelle Gallagher, DO (Faculty).

**ANTIBIOTIC DISCONTINUATION IN NON-ICU HOSPITALIZED PATIENTS WITH RESPIRATORY TRACT INFECTIONS HAVING LOW PROCALCITONIN VALUES. Andrew Victor, MD (Resident);** Kaitlyn Zurek, MD (Faculty).

**THE IMPACT OF BREASTFEEDING ON THE NEONATAL ADAPTION OF INFANTS EXPOSED TO SSRIS AND SNRIS IN UTERO. Torrey Halbert, DO (Resident);** Robin Blumer, MD (Faculty).

**MESTERIOUS RENAL MASSES. Ross Voelker, DO (Resident);** Kathik Ranganathan, DO (Resident); Richard Sarle, MD (Faculty).

**PURULENT PERICARDITIS WITH TAMPONADE DUE TO *H. INFLUENZAE* IN AN IMMUNE COMPETENT HOST. Andrew Good, DO (Resident);** Mckalin Cox, DO (Resident); Alyssa Wood, DO (Resident); Christopher Nedzlek, MD (Faculty).

**FROM TUSSIS TO TESTICULAR PAIN, A CASE REPORT ON ACUTE IDIOPATHIC INTRATESTICULAR HEMORRHAGE. Alexander Haberman, DO (Resident);** Eric Stockall, MD (Faculty).

**IFR6 EFFECT ON ORAL ADHESIONS. Oshadi Caldera, BSc (Medical Student);** Paula Somohona; Saira Malik, PhD; Brian C Schutte, PhD (Faculty).

**INFLUENZA B-ASSOCIATED HELLP SYNDROME COMPLICATING PREGNANCY: A CASE REPORT. Calley Gober, DO (Resident);** Katherine Betcher, MD (Faculty); Aly Bowen (Medical Student).

**ABDOMINAL SCHWANNOMA UNVEILING LYMPHOMA: A RARE ASSOCIATION IN A COMPLEX CLINICAL PRESENTATION. Peter Ng, MD (Resident);** Varisha Singh, MD (Resident); Ayesha Oaris, MD (Resident); Meggan Robinson, DO (Faculty).

**INSIGHTS OF HIGHER ORDER MODELLING OF DIFFUSION MAGNETIC RESONANCE IMAGING IN BRAIN INJURY PATIENTS WITH A GLASGOW COMA SCALE OF 13-15: A TRACK-TBI STUDY. Joshua H. Baker, MS (DO/PhD Candidate);** Andrew Bender, PhD (Faculty); Norman Scheel, PhD (Faculty); David C. Zhu, PhD (Faculty); The TRACK-TBI Investigators.

**ENHANCING PATIENT SATISFACTION IN EMERGENCY MEDICINE: A QUALITY IMPROVEMENT INITIATIVE TO IMPROVE PRESS GANEY SCORES THROUGH PHYSICIAN EDUCATION AND COMMUNICATION STRATEGIES. Ayman Alhadheri, MD (Resident);** Ilana Tidus, DO (Resident); Christian Whittier, DO (Resident); Olga J. Santiago-Rivera, PhD (Faculty); Nikolai Butki, DO (Faculty); Martina Ghiardi, DO (Faculty); Abdullah A.B. Bokhari, DO (Faculty).

**DRUG-INDUCED SLEEP ENDOSCOPY FOR TARGETED SLEEP SURGERY IN PEDIATRIC PATIENTS. Ani Mnatsakanian, DO (Resident);** Theresa Schneider, DO (Resident); Anya Costeloe, DO (Resident); Jacob Surma (Medical Student); Suzanne Forman, DO (Resident); Michael Haupert, DO (Faculty); Prasad John Thottam, DO (Faculty).

**FROM ANXIETY TO NEURONAL RESCUE: EXPLORING THE POTENTIAL OF LORAZEPAM IN TRAUMATIC BRAIN INJURY. Luke Noah, BSc (Medical Student);** Alex Pasculle (Medical Student); Siva Koduvayur (Medical Student); Charles C. Hong, MD, PhD (Faculty); Charles H. Williams PhD (Faculty).

**TRANSVERSE MYELITIS IN PEDIATRICS: A CASE STUDY. Nathan Fleischman, MD (Resident);** Eric Kucel, DO; Kristen Owen, DO; Pamela Emmanuel, MD (Faculty).

**CELIAC DISEASE PRESENTING WITH DUODENAL ULCERATION. Mark Rigby, DO (Resident);** Brandon Wiggins, DO (Fellow); Manthan Patel (Medical Student); Mark Minaudo, DO (Faculty).

**PURE MATURE TERATOMA IN POST-PUBERTAL TRANSGENDER FEMALE. Mercedes Kent, DO (Resident);** Robert Dimitriou, MD (Faculty). xx

**ACUTE KIDNEY INJURY INDUCED BY RHABDOMYOLYSIS IN A YOUNG MALE WITH PREVIOUSLY UNDIAGNOSED HYPOTHYROIDISM. Sushmita Mantravadi, MD (Resident);** Apoorv Tiwari, MD (Resident); Sujata Kambhatla, MD (Faculty).

**HEAD AND NECK COOLING AS AN EMERGENCY DEPARTMENT THERAPY TO DECREASE PROGRESSION OF CONCUSSIVE SYMPTOMS: A PILOT STUDY. Daniel Mattox, DO (Resident);** Danielle Lorenz, DO (Resident); Matthew Hysell, MD (Faculty); Monique Luna, MS.

**QUALITY IMPROVEMENT: IMPROVING ANTIBIOTIC STEWARDSHIP IN COMMUNITY-ACQUIRED PNEUMONIA. Raagini Suresh Yedidi, MD (Resident)**; Aaron Gandhi, DO (Resident); Apoorv Tiwari, MD (Resident); Kashif Khan, MD (Resident); Sujata Kambhatla, MD (Faculty); Amin Pasha, MD (Faculty).

**A CASE REPORT OF UPPER EXTREMITY DVT PRESENTING AS METASTATIC LYMPHOMA. Gretchen Yormick, MS (Medical Student);** Heba Al Sabbagh, MD (Resident); Lara Bitar, MD (Resident); Mohammad Saleh, MD (Faculty).

**TRAUMA OBSCURING LEUKEMIA - A UNIQUE CASE REPORT**. **Celine Adriano, BS (Medical Student);** Spencer Gilbert, BS (Medical Student); Harika Doddi, MD (Resident); Ammar Hatahet, MD, MPH, FACP (Faculty).

**INVESTIGATING VARIANTS OF TUMOR SUPPRESSOR GENES IN HISTIOCYTIC SARCOMA**. **Yutong Liang (Medical Student)**; Vilma Yuzbasiyan-Gurkan, PhD (Faculty); Ya-ting Yang, DVM (Postdoctoral); Alex Engleberg (PhD Student).

**SURVEY STUDY: SPORTS INJURIES IMPACT ON MENTAL HEALTH, ACADEMICS, AND INTERPERSONAL RELATIONSHIPS IN COLLEGIATE ATHLETES. Landon Barlow (Medical Student);** Aashir Siddique (Medical Student); Nathan Fitton, DO, (Faculty).

**UNCOVERING NOVEL EPIGENOMIC SIGNATURES IN HPV AND NON-HPV HEAD AND NECK SQUAMOUS CELL CARCINOMA**. **Christian Gluck, MD, PhD (Resident);** Akinsola Oyelakin, PhD (Graduate Student); Alexandra Glathar, PhD (Graduate Student); Michael Buck, PhD (Faculty); Rose-Anne Romano, PhD (Faculty); Satrajit Sinha, PhD (Faculty).

**USING SMALL MOLECULES TO IDENTIFY CRITICAL HOST-CELLULAR PATHWAYS FOR BRUCELLA INFECTION**. **Thomas Kim, BA (DO/PhD Student);** Erika Lisabeth, PhD (Faculty); Richard Neubig, MD, PhD (Faculty); Bill Hong, PhD (Faculty); Thomas O’ Halloran, PhD (Faculty); Aretha Fiebig, PhD (Faculty); Sean Crosson, PhD (Faculty).

**CONGENITAL AGENESIS OF THE INFERIOR VENA CAVA IN THE SETTING OF PRESENTING PULMONARY EMBOLISM: A CASE STUDY**. **Victoria Brown, DO (Resident);** Suruchi Dash, DO (Resident); David Betten, DO (Faculty).

**CASE REPORT: GUILLAIN-BARRE SYNDROME, AN UNUSUAL PRESENTATION**. **Ania Pathak, PhD (DO/PhD Student**), Cire Nemeth, DO (Resident).

**CONDITIONAL DELETION OF TBX4/5 CAUSES PULMONARY HYPERTENSION THROUGH DISRUPTED EPITHELIAL, VASCULAR, AND SMOOTH MUSCLE DEVELOPMENT**. **Kaylie Chiles, BS, BS, MS (DO/PhD Student);** Madeline Dawson, BS (Research Assistant); Ripla Arora, PhD (Faculty & PI).

**CASE REPORT REVIEW HIGHLIGHTING AN OUTPATIENT APPROACH IN ATYPICAL DIABETIC PRESENTATION IN YOUNG ADULT**. **Marie Kenny, DO (Resident);** Michelle Barbat, DO (Resident); Jeremy Fischer, DO (Faculty).

**CLASSIC ERYTHEMA MIGRANS RASH IN PEDIATRIC PATIENT WITH DISSEMINATED LYME DISEASE AND CONCERN FOR LYME MENINGITIS**. **Brandon Popp, DO (Resident);** Jeffrey Summers Jr. (Resident), MD; Ghufraan Akram, MD (Resident); Kristen Owen, DO, FACEP (Faculty).

**STREPTOCOCCUS VIRIDANS NECROTIZING FASCIITIS PRESENTING AS FOURNIER GANGRENE IN A DIABETIC PATIENT**. **Aaron Gandhi, DO (Resident**); Apoorv Tiwari, MD (Resident); Ali Jishi, MD (Resident); Sujata Kambhatla, MD (Faculty), Amin Pasha, MD (Faculty).

**A CASE REPORT OF DAPAGLIFLOZIN ASSOCIATED PANCREATITIS**. **Chaoneng Wu, MD (Resident);** Abed Dabaja, MD (Faculty).

**MEDICAL STUDENT EXPERIENCES WITH PELVIC EXAMS UNDER ANESTHESIA: A SCOPING REVIEW**. **Varsha Reddy (Medical Student);** Sarah Lynch (Medical Student); Mahi Basra (Medical Student); Nicholas Blackmond (Graduate Student); Sheena Maldonado (Undergraduate Student); Angela Fleming, DO (Faculty).

**NO DIFFERENCE IN FINAL RADIOGRAPHIC FRACTURE DISPLACEMENT IN TIBIAL PLATEAU FRACTURES: IMN VS ORIF**. **Adrian Olson, DO (Resident)**; Ivan Bandovic, DO (Resident); Ryan Centanni, BS (Medical Student); Austin Smith, BA (medical Student); Virginia Leadbetter, BS (Medical Student); Alan Afsari, MD (Faculty); Benjamin Best, DO (Faculty).

**DEVELOPMENTAL EXPOSURE TO THE PARKINSON’S DISEASE-ASSOCIATED ORGANOCHLORINE PESTICIDE DIELDRIN ALTERS DOPAMINE NEUROTRANSMISSION IN A-SYNUCLEIN PRE-FORMED FIBRIL (PFF)-INJECTED MICE**. **Sierra L. Boyd,** Nathan C. Kuhn; Joseph R. Patterson; Anna C. Stoll; Sydney A. Zimmerman; Mason R. Kolanowski; Joseph J. Neubecker; Kelvin C. Luk; Eric S. Ramsson; Caryl E. Sortwell; Alison I. Bernstein.

**AN INTERESTING YET UNCOMMON PRESENTATION OF SENSORINEURAL HEARING LOSS DUE TO HYPOTHYROIDISM. Suhitha Bysani, MD (Resident)**; Amna. N. Kiani, MD (Resident); Naisargee Solanki, MD (Resident); Mohammed Bilal, MD (Resident); Ahmed. J. Chaudhary, MD, FACP (Faculty).

**NONINVASIVE MANAGEMENT OF INTRACTABLE HICCUPS**. **Derek Bowman (Medical Student)**; Rozzie Bloch; Megan Kempa; Canyon O’Brien; Kayla Fong; Ethan Poland.

**ANEMIA, MELENA, AND AN INCONCLUSIVE BIOPSY: A CHALLENGING CECAL MASS CASE. Gillian Tyner, DO (Resident**); Abu Fazal Shaik Mohammed, MD (Faculty); Ahmad H Aburashed, MD (Faculty).

**CHARACTERIZATION OF FISH AND REPTILIAN PARVALBUMINS AS RELEVANT FOOD ALLERGENS**. **Andrea O’Malley, PhD (Postdoctoral Research Associate);** Thimo Ruethers, PhD (Faculty); Andreas Lopata, PhD (Faculty); Joshua M. Ray, BS; Tomasz Cierpicki, PhD (Faculty); Krzysztof Kowal, MD, PhD (Faculty); Maksymilian Chruszcz, PhD (Faculty).

**STERCORAL COLITIS DUE TO PICA SYNDROME IN A YOUNG ADULT: A CASE REPORT**. **Anh Tuan Mai, MD (Resident);** Faraz Mahmood, MD (Resident); Raied Hanna, MD, FACP (Faculty).

**MANDIBULAR OSTEITIS FIBROSA CYSTICA: AN UNUSUAL MANIFESTATION OF PRIMARY HYPERPARATHYROIDISM**. **Gabriele Noreikaite, DO (Resident);** Marwan Boulis, MD (Faculty).

**POPLITEAL ARTERY ARTERIOVENOUS FISTULA FOLLOWING KNEE ARTHROSCOPIC SURGERY**. **Harminder Sandhu, MHSc (Medical Student);** Sonal Kaushik, DO (Resident); Micheal Hudson, MD (Resident); John Iljas, DO (Faculty).

**A COMPREHENSIVE CASE REPORT OF SEVERE PELVIC INFLAMMATORY DISEASE WITH EXTENSIVE TUBO-OVARIAN AND INTRA-ABDOMINAL ABSCESSES**. **Mackenzie Miller, BS (Medical Student);** Sharmila Nasrin, BSN (Medical Student).

**CAROTID SINUS SYNDROME IN A PATIENT WITH ADVANCED LARYNGEAL CANCER. Montaser Elkholy, MD (Resident**); Naisargee Solanki, MD (Resident); Zainab Muslehuddin, MD (Faculty).

**CIPROFLOXACIN-INDUCED NEUROTOXICITY, A RARE PRESENTATION. Sumit Gami, MD (Resident);** Zainab Muslehuddin, MD (Faculty).

**A CASE REPORT OF TREATMENT-INDUCED PULMONARY FIBROSIS IN A 68-YEAR-OLD FEMALE WITH PLATINUM-RESISTANT EPITHELIAL OVARIAN CARCINOMA. Rakesh Shah, MD (Resident);** Priyanka Bhagat, MD (Resident); Khaled Harmouch, MD (Resident); Sumit Gami, MD (Resident); Kauser Hafeez, MD, MPH (Resident); Ahmed J Chaudhary, MD, FACP (Faculty).

**MANIPULATING THE BLOOD LABYRINTH BARRIER WITH MANNITOL TO PREVENT CISPLATIN-INDUCED HEARING LOSS**. **Ayesha Noman (Medical Student**); Subhendu Mukherjee, PhD; Trung Le, MD, PhD, FRCSC.

**DELETION OF EPE1 GENE ENHANCES LONGEVITY IN SCHIZOSACCHAROMYCES POMBE**. **Yongqi Xu (Medical Student);** Tommy Vo (PhD, Faculty).

**DELIRIUM TREMENS IN A MIDDLE-AGED MALE ICU PATIENT**. **Yousef Alsmairat, MD (Resident**); Zainab Muslehuddin, MD (Faculty).

**A DIAGNOSTIC DILEMMA FOR A CHRONIC BLISTERING DISORDER**. **Ishani Singh, MD (Resident);** Zainab Muslehuddin, MD (Resident); Sumit Gami, MD (Resident); Malitha Hettiarachchi, MD (Faculty).

**COMPARATIVE ANALYSIS OF ANTHROPOMETRIC MEASUREMENTS OF CHILDREN IN UNDERSERVED COMMUNITIES OF GUATEMALA AND DOMINICAN REPUBLIC.** E**mma Schaefer, (Medical Student)**^1^; Maria Rollinger, (Medical Student);^2^ Emily Ubbens, (Medical Student);^1^ Rene Hinojosa, PhD (Faculty).^1,3^

**A RARE CASE OF ACUTE PULMONARY EMBOLISM MASQUERADING AS ACUTE MYOCARDIAL INFARCTION.Karishma Ashwin Doshi, MD (Resident);** Apoorv Tiwari, MD (Resident); William Harder, DO (Faculty); Sujata Kambhatla, MD (Faculty).

**TYROSINE KINASE INHIBITOR INDUCED PERICARDIAL EFFUSION IN ISOLATION**. **Stephen Caucci, DO (Resident);** Nicholas Campbell, DO (Fellow); David McComb, DO (Faculty).

**FATAL SEPTIC SHOCK DUE TO WEEKSELLA VIROSA IN A 69-YEAR-OLD MALE**. **Baral Dilip, MD (Resident**); Messina Alvarez Angelo, MD (Resident); Hafeez Wasif, MD (Faculty).

**INVESTIGATING GROUP B STREPTOCOCCUS (GBS) GENETIC FACTORS ASSOCIATED WITH VERTICAL TRANSMISSION AND INVASIVE NEONATAL DISEASE IN NIGERIA**. **Jade E. Le Bris (DO/PhD Student);** Macy E. Pell, PhD (Post-doc); Nubwa Medugu, MD (Faculty); Shannon D. Manning, PhD, MPH (Faculty).

**FAMILIAL DYSPHAGIA AND ITS ASSOCIATION WITH EOSINOPHILIC ESOPHAGITIS**. **Ananya Varre (Medical Student);** John P. Karns (Medical Student); Emily Ciokajlo (Medical Student); Gustav Westerlund, MBA (Faculty); Furqan Irfan, MBBS, PhD (Faculty).

**ADHESIVE CAPSULITIS EMBOLIZATION: A TECHNIQUE FOR THE FUTURE**. **John P. Karns (Medical Student);** Arif Musa, MD (Resident); Foaz Kayali, MD, PhD (Faculty); Ali Harb, MD (Faculty).

**COMPARISON OF ANTHROPOMETRIC MEASUREMENTS OF CHILDREN LIVING IN THE DOMINICAN REPUBLIC AND PERU**. **Zack Murphy (Medical Student);** Megan Thomas (Medical Student); Melissa Xu (Medical Student); Rene Hinojosa, PhD (Faculty).

**A CASE OF ASYMPTOMATIC AORTA-ATRIAL FISTULA**. **Nicholas Campbell, DO (Fellow);** Majid Mughal, MD (Faculty).

**EXPLORING THE EFFECTS OF TRAUMA ON ADOLESCENT BRAIN AGING: A PRELIMINARY NEUROIMAGING STUDY**. **Shravya Chanamolu (Medical Student);** Ahmad Almaat (Undergraduate Student); Reem Tamimi (Graduate Student); Carmen Carpenter (Research Assistant); Samantha L. Ely (PhD Student); Leah Gowatch (Research Assistant); Jovan Jande (Medical Student); MacKenna Shampine (Research Assistant); Clara G. Zundel (Postdoctoral Research Fellow); Hilary A. Marusak (Principal Investigator).

**“ARE YOU BREASTFEEDING?” A SURVEY STUDY ON CAREGIVERS PERCEPTIONS OF HEALTHCARE PROVIDERS LEVEL OF SUPPORT FOR INFANT FEEDING METHODS:, PRESSURE TO BREASTFEED AND CAREGIVERS EMOTIONAL RESPONSE**. **Maya Wyrzykowski (Medical Student);** Dale D’Mello, MD (Faculty); Ethan Dawson-Baglien, PhD, D.O. (Resident).

**UNCOMMON JEJUNOJEJUNOSTOMY PERFORATION: A CASE STUDY ON COMPLICATIONS FOLLOWING ROUX-EN-Y GASTRIC BYPASS**. **Stephanie Agudelo (Medical Student)**; Emily Mathjis (Medical Student); Abubaker Ali, MD, FACS.

**EMERGENT PRESENTATION OF AN OOCYTE RETRIEVAL COMPLICATION**. **Garrett Tomaski, MD (Resident);** Jacob Gylten, MD (Resident); Alan Lazzara, MD (Faculty).

**A UNIQUE FINDING – MELANOSIS OF THE BLADDER**. **Lauren Nesi (Resident);** Parmjot Gogia (Graduate Student); Dongping Shi (Faculty); Barrett Anderson, DO (Faculty).

## 01

## Abstract

The Spartan Medical Research Journal (SMRJ) is pleased to publish abstracts from the Second Annual Research Day hosted by the Michigan State University College of Osteopathic Medicine (MSUCOM), held in Novi, Michigan, on April 11, 2024. Sponsored by MSUCOM, the Statewide Campus System (SCS), and Research, Innovation, and Scholarly Engagement (RISE), this event showcased a total of 148 selected research abstracts following a meticulous blinded review by the MSUCOM Research Day Planning Committee and SMRJ editorial staff. These abstracts were subsequently presented at the MSUCOM Second Annual Research Day in 2024, with awards for exceptional oral and poster presentations conferred on April 11, 2024. Of the 148 presentations that were ultimately chosen, 98 authors consented and elected to have their abstracts published in SMRJ.

The selected abstracts encompass a wide array of contemporary medical and clinical subjects, incorporating a variety of research designs that cover basic science, clinical research, case reports, medical education, and quality improvement projects. While these abstracts offer a concise overview of the research projects or presentations, they do not permit a comprehensive evaluation of the scientific rigor employed in the respective works. Although these abstracts offer preliminary results that may necessitate further refinement and validation, they serve a vital function in disseminating novel research concepts and advancements in the discipline of medicine. This knowledge-sharing promotes meaningful dialogue among researchers, clinicians, and educators, thereby making a valuable contribution to the collective body of knowledge in the fields of medical sciences and osteopathic medicine.

Authors are strongly encouraged to pursue the publication of their research as full manuscripts. The MSUCOM Research Day Committee extends gratitude to all those who submitted abstracts for consideration, as we anticipate another exceptional season of submissions for MSUCOM Research Day 2025.

Andrea Amalfitano, DO, PhD

Osteopathic Heritage Foundation Professor of Pediatrics, Microbiology and Molecular Genetics

Professor, BioMolecular Science Gateway

Editor-in-Chief, Spartan Medical Research Journal (SMRJ)

MSU College of Osteopathic Medicine- Statewide Campus System

C. Patricia Obando S., PhD.

Associate Dean and DIO, Graduate Medical Education

Associate Professor- MSU College of Osteopathic Medicine- Statewide Campus System

Rana Ismail, PhD, MSc, CPHQ

Director of Research

Editor, Spartan Medical Research Journal (SMRJ)

MSU College of Osteopathic Medicine- Statewide Campus System

Francis Akenami, BMLS, PhD, MSc, FIMLS

Managing Editor

Spartan Medical Research Journal (SMRJ)

MSU College of Osteopathic Medicine- Statewide Campus System

